# Supplementary material for: Distinct neural sources underlying visual word form processing as revealed by steady state visual evoked potentials (SSVEP)
Source: Sci Rep. 2021 Sep 14;11:18229. doi: 10.1038/s41598-021-95627-x (PMC8440525; doi:10.1038/s41598-021-95627-x)
Supplement: Supplementary file 1 — Supplementary Information. [file 41598_2021_95627_MOESM1_ESM.pdf]

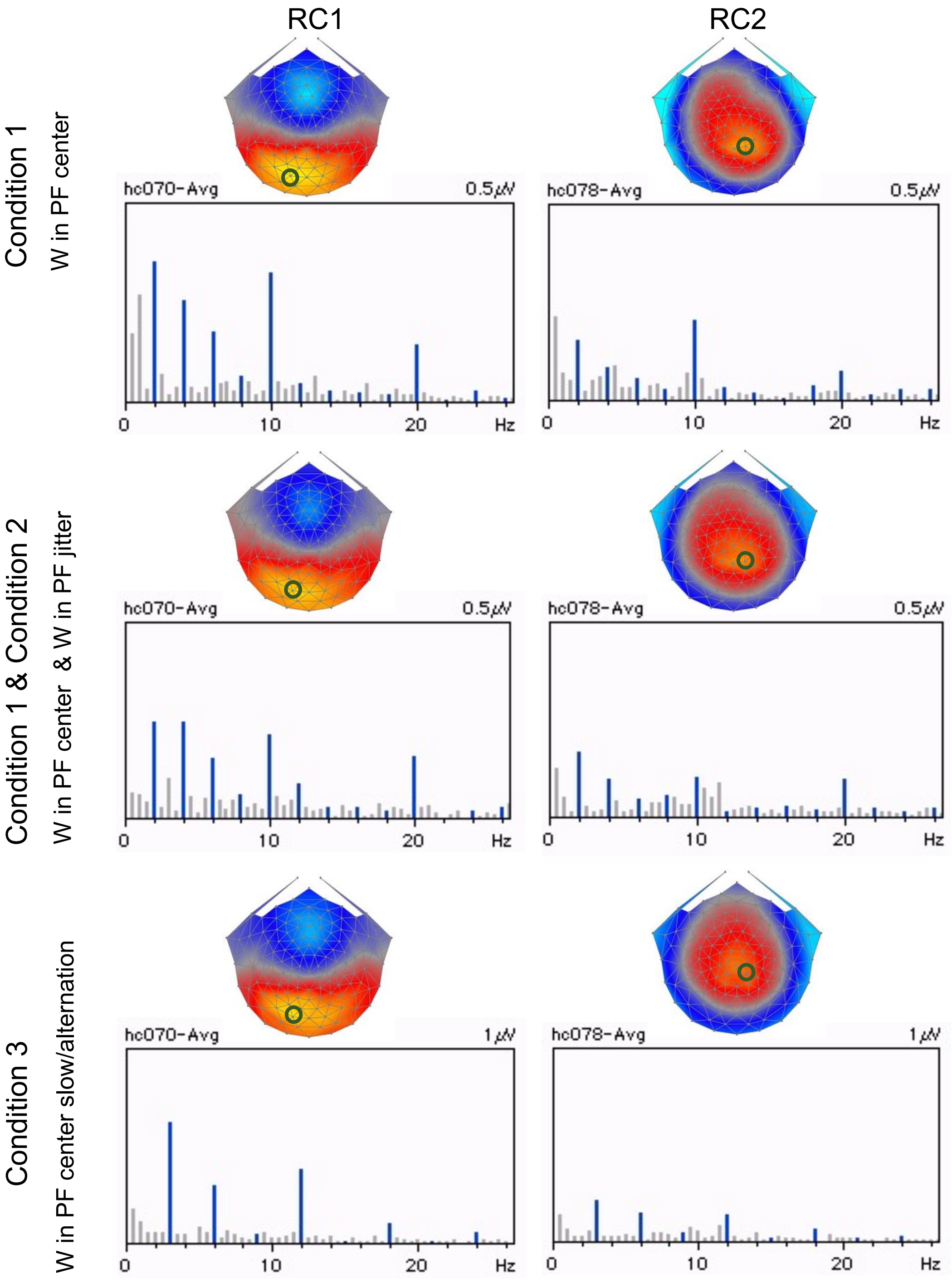

Supplementary Figure 1. We picked representative sensors near the peak value of RC1 and RC2 (i.e., EGI sensor 70 and 78, respectively) and illustrated their EEG spectra across both frequencies that represent the base and deviant stimulation rates, their harmonics, as well as intermediate frequencies which were unrelated to the respective stimulation rates. Blue lines show responses at each relevant stimulation frequency and the related integer harmonics (i.e., 2Hz, 4Hz, 6Hz, 8Hz, 10Hz … in conditions 1 and 2; 3Hz, 6Hz, 9Hz, 12Hz, 15Hz … in condition 3). The grey lines represent background EEG activity not related to the stimulation frequencies.
